# Improved accuracy and fewer outliers with a novel CT-free robotic THA system in matched-pair analysis with manual THA

**DOI:** 10.1007/s11701-021-01315-3

**Published:** 2021-10-28

**Authors:** Atul F. Kamath, Sridhar M. Durbhakula, Trevor Pickering, Nathan L. Cafferky, Trevor G. Murray, Michael A. Wind, Stéphane Méthot

**Affiliations:** 1 Orthopaedic and Rheumatologic Institute, Cleveland Clinic, 9500 Euclid Avenue, A40, Cleveland, OH 44195 USA; 2 Washington Joint Institute, Bethesda, MD 20817 USA; 3 Mississippi Sports Medicine and Orthopaedic Center, Jackson, MS 39202 USA; 4 Vail Summit Orthopaedic Foundation, Vail, CO 81657 USA; 5 OrthoVirginia, Richmond, VA 23235 USA; 6 Zimmer CAS, Montréal, QC H3C-2N6 Canada

**Keywords:** Hip arthroplasty, Robotic surgery, Inclination, Version, Leg length discrepancy, Accuracy

## Abstract

Accurate component orientation and restoration of hip biomechanics remains a continuing challenge in total hip arthroplasty (THA). The goal of this study was to analyze the accuracy/reproducibility of a novel CT-free and pin-less robotic-assisted THA (RA-THA) platform compared to manual THA (mTHA). This matched-pair cadaveric study compared this RA-THA system to mTHA (*n* = 33/arm), both using the assistance of fluoroscopic imaging, in a group of 14 high-volume arthroplasty surgeons. In both groups, surgeons were asked to aim for 40°/15° for cup inclination/version, and 0 mm of leg length discrepancy (LLD). A validated and accurate method using radio-opaque markers measured cup inclination/version and LLD. The accuracy and reproducibility (fewer outliers) of cup inclination was significantly improved in the robotic group (1.8° ± 1.3° vs 6.4° ± 4.9°, respectively, robotic vs manual; *p* < 0.001), with no significant difference between groups for version. The reproducibility of LLD was significantly improved in the robotic group (*p* = 0.003). For all parameters studied, the robotic group had an improved accuracy and lower variance (fewer outliers). The percentage of cases within the more restrictive Callanan safe zone was 100% for RA-THA vs 73% for mTHA (*p* = 0.002). The CT-free RA-THA platform, using only fluoroscopic imaging, demonstrated more accurate acetabular cup positioning, when compared to the mTHA procedures performed by high-volume hip surgeons (naive to this RA-THA platform), with respect to cup inclination and placement within the Lewinnek/Callanan safe zones. Future study must incorporate economic factors, lower volume surgeons, clinical and patient-centric outcomes, and other radiographic parameters in controlled studies in large sample sizes.

## Introduction

While total hip arthroplasty (THA) is a highly successful orthopedic intervention, proper component orientation and restoration of hip biomechanics remain a continuing challenge. Complications such as dislocation [1], impingement [2], liner wear [3], altered gait mechanics [4], leg length discrepancy (LLD) [4], and failure requiring revision surgery [5] all relate to accurate THA implantation.

Robotic assistance in hip arthroplasty has gained increasing interest to improve the accuracy of component positioning [6, 7], but also for real-time data feedback to the surgeon. Studies comparing the radiologic outcomes of robotic arm assisted (RA-THA) versus manual total hip arthroplasty (mTHA) have included cohorts utilizing both semi-active [8–14] and fully active [15, 16] robotic systems. Commercially available robotic systems require pre-operative computed tomography (CT), navigation pins, and/or significant changes from the intraoperative workflow when compared to manual surgery. This presents challenges for both surgical efficiency, as well as the overall costs attributable to the robotic technology.

To address the challenges associated with contemporary robotic systems, a novel robotic THA platform was developed. The system does not utilize CT nor intraoperative optical navigation guidance (no bone tracker pins/arrays). Rather, robotic-assisted acetabular component placement and digital information related to restoration of key biomechanical and component parameters is generated by fluoroscopic imaging alone, with the ability to link pre-operative templating and post-operative data synthesis.

The goal of the study was to analyze radiologic outcomes between RA-THA and mTHA groups in a group of high-volume arthroplasty surgeons: (1) the accuracy of acetabular component orientation (inclination, version, and percent within safe zones); and (2) the ability to equalize radiographic LLD.

## Materials and methods

### Study design

This matched-pair study was conducted on 66 hips in 33 cadaveric specimens (18/15 males/females) with a mean age of 79 ± 9 years (range 59–91). For each specimen, one hip was randomly assigned to the robotic group and the contralateral to the manual group. The G7^®^ Acetabular system, and compatible femoral implant systems: Avenir^®^, Avenir Complete™, Taperloc^®^ Complete, Echo Bi-Metric^®^ and Echo Bi-Metric Microplasty^®^ (Zimmer Biomet, Warsaw IN, USA), were implanted in both groups, with the same implant combination in both limbs of the specimen.

Fourteen board-certified arthroplasty surgeons each performed direct anterior approach THA procedures on 2–3 specimens using robotic and manual instrumentation, both with the assistance of fluoroscopic imaging. With respect to direct anterior approach experience, most surgeons (12/14) were considered high volume, with a minimum of 175 procedures/year (range 175–810). The remaining two surgeons perform 175–200 procedures/year using either a posterior or anterolateral approach.

### Intraoperative CMM acquisitions

**Fig. 1:**
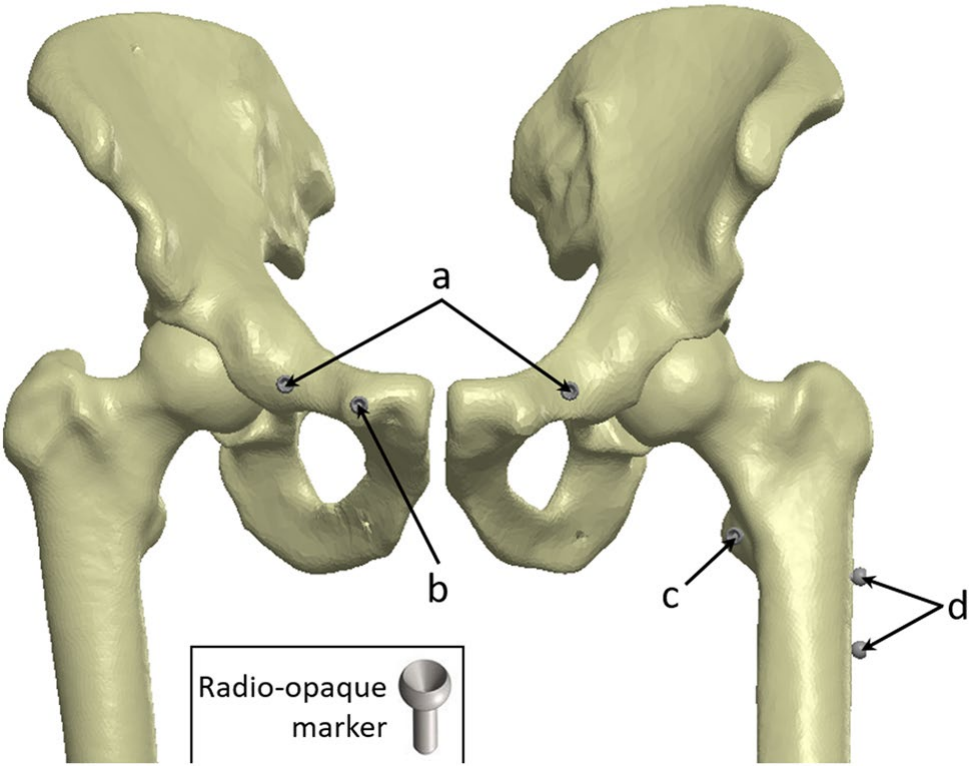
Location of radio-opaque markers for CMM acquisitions (example of a left hip: 6 markers). Markers (insert) were manufactured and inspected to make sure they were within specifications. Making sure not to interfere with the THA incision, they were inserted press-fit into a pre-drilled hole in the bone at the following locations: **a** illiopectineal eminence, aligned over each teardrop (named ipsilateral and contralateral teardrop); **b** pelvis reference; **c** lesser trochanter; and **d** proximal and distal femoral axis. Markers were used to validate the accuracy of the RA-THA platform, and are not part of the clinical use of the system

A calibrated CMM (coordinate measurement machine; Quantum FaroArm^®^, Lake Mary FL, USA; maximum error 0.05 mm) with a 1/8'' extended ball probe was used to acquire intraoperative accuracy data using radio-opaque markers positioned in each specimen by personnel specifically trained on the CMM (Fig. 1). At different steps during the surgical flow, the procedure was halted to perform the CMM acquisitions (Table 1). The CMM measurements using radio-opaque markers were used to validate the accuracy of the RA-THA platform, and are not part of the clinical use of the system.

**Table 1:** Intraoperative CMM acquisitions^a^

#	Surgical step	Parameter	CMM acquisitions^b^
1	Before direct anterior approach THA incision	Initial leg length	C-arm detector plane (3 points distributed on the surface) Ipsilateral and contralateral teardrop radio-opaque markers Pelvis reference radio-opaque marker Lesser trochanter radio-opaque marker Proximal and distal femoral axis radio-opaque markers
2	After hip dislocation/femoral head removal. Before reaming	Initial femoral COR^c^	Ipsilateral and contralateral teardrop radio-opaque markers Pelvis reference radio-opaque marker Acetabular wall (12 points, avoiding acetabulum fossa)
3	After acetabular component impaction	Cup orientation	C-arm detector plane (3 points distributed on the surface) Ipsilateral and contralateral teardrop radio-opaque markers Cup rim plane (3 points distributed on the surface)
4	Reduced joint with final implant components	Final leg length	Same as step #1
5	Perform ultimate hip dislocation to expose the acetabular component	Final femoral COR	Ipsilateral and contralateral teardrop radio-opaque markers Pelvis reference radio-opaque marker Interior of acetabulum component liner (12 points)

^a^The CMM acquisition of radio-opaque markers was used to validate the RA-THA platform; CMM and associated markers are not part of the clinical use of the RA-THA system

^b^All acquisitions were performed in triplicates

^*c*^*COR* center of rotation

### Manual THA procedure

Surgeons performed a direct anterior approach THA in accordance with the most current surgical technique of the implant system used, using their preferred instruments and surgical workflow, and with the assistance of fluoroscopic imaging. Surgeons were asked to aim for 40°/15° of inclination/version for the acetabular component orientation, and 0 mm of LLD. Fluoroscopic guidance was used to establish a leveled pelvis (i.e., symmetrical obturator foramina), evaluate bone preparation, position the components, and equalize LLD.

### Robotic THA procedure

**Fig. 2:**
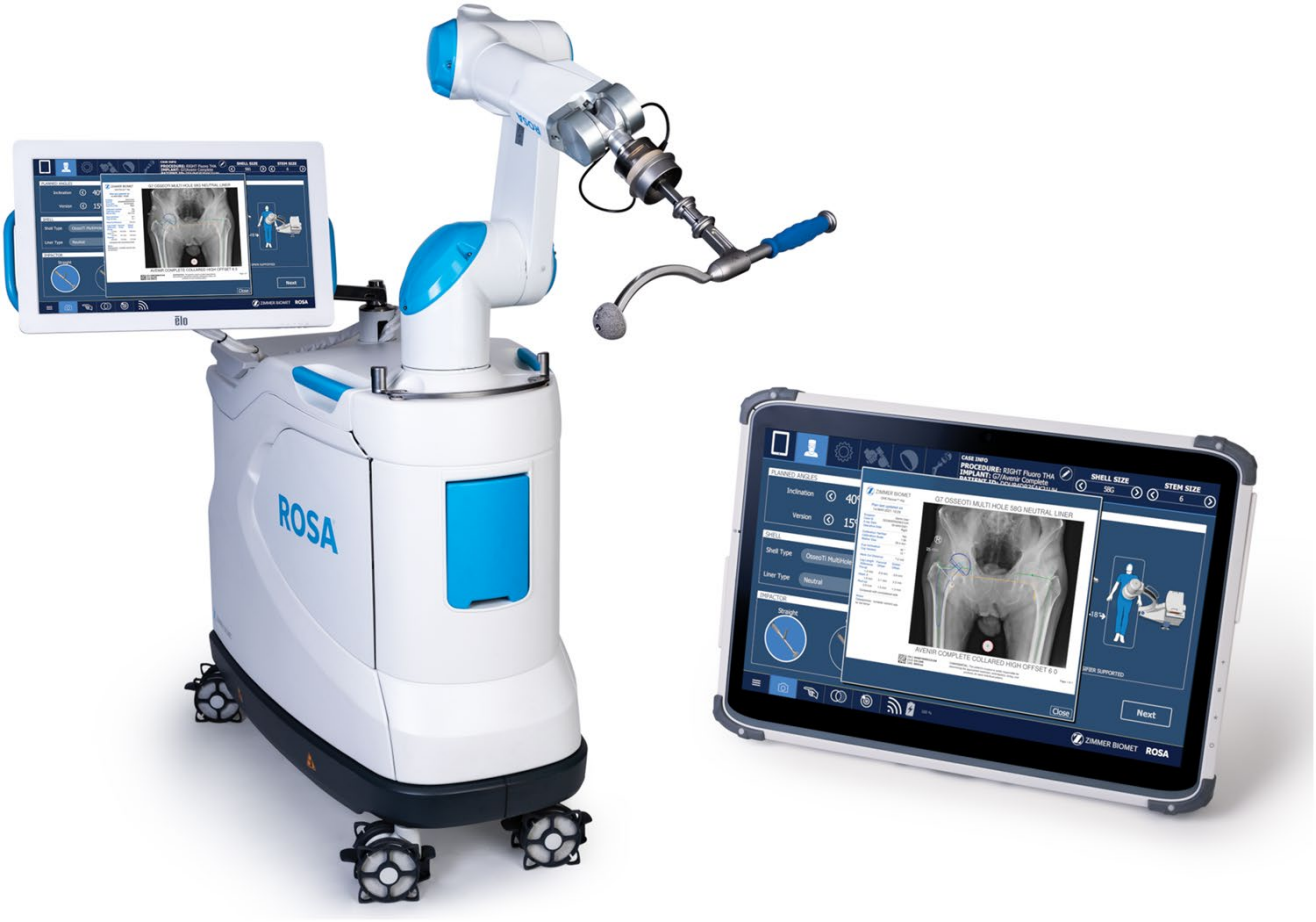
ROSA^®^ Hip System comprising the ROSA^®^ Recon Robotic Unit and the ROSA^®^ Tablet. The preparation steps of ROSA^®^ Hip include: **1** connecting the tablet to the robotic unit using Wi-Fi; **2** selecting or reviewing (if a pre-operative plan was completed) surgical parameters such as planned angles, measurements, shell and stem type, impactor and C-arm diameter; **3** installing the quick connect interface at the end of the robotic arm; **4** draping the robotic arm and robotic unit; and **5** calibrating the force sensor

Given the novelty of this application, none of the surgeons had experience on the ROSA^®^ Hip System (Zimmer Biomet, Warsaw IN, USA; Fig. 2). All surgeons received standardized training consisting of theoretical and hands-on surgical training on sawbones. As with the manual group, surgeons were asked to target 40°/15° of inclination/version, and 0 mm of LLD.

**Fig. 3:**
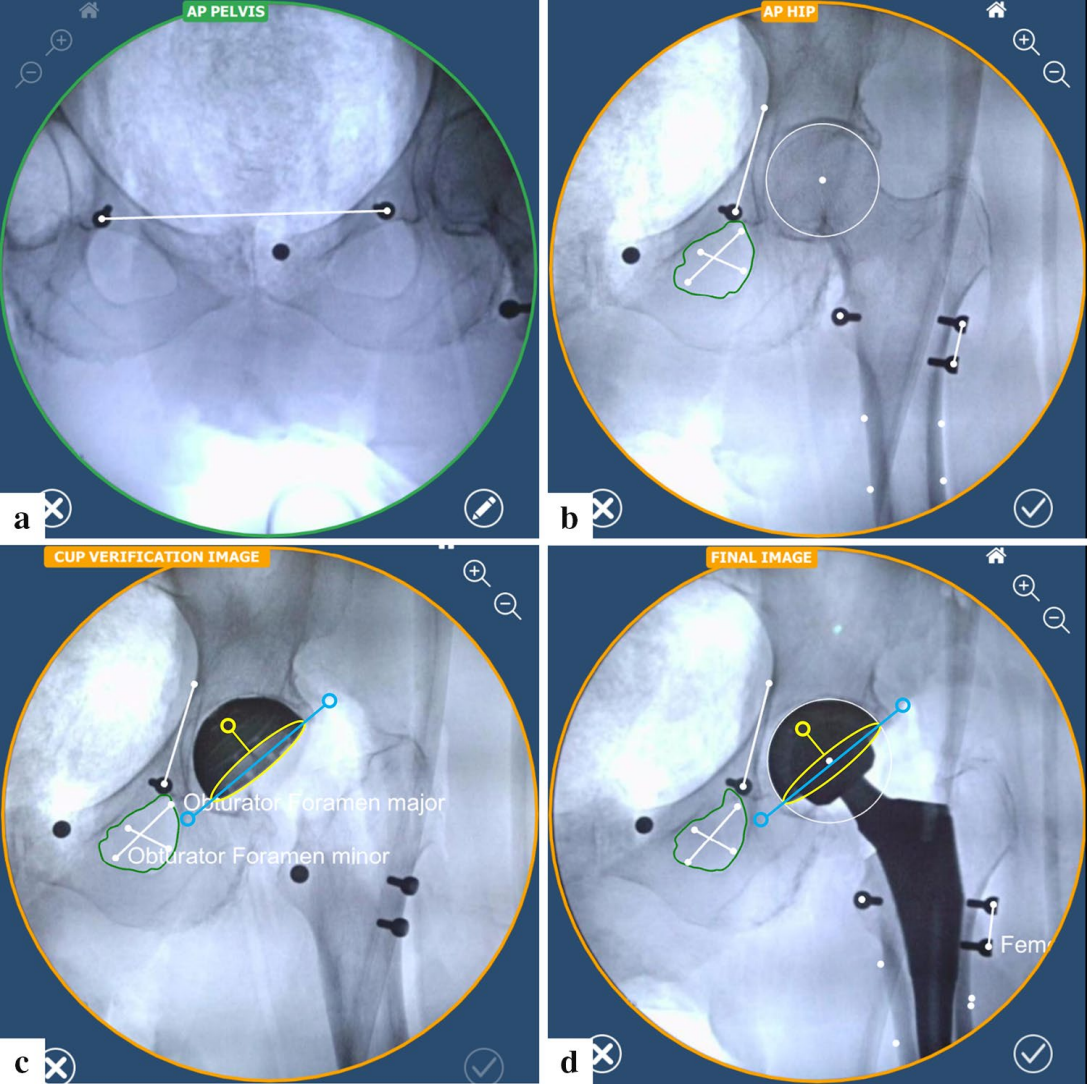
Positioning of the landmarks. **a** Pelvis reference image: ipsilateral and contralateral teardrop markers. **b** Hip reference image: the ipsilateral and contralateral teardrop, lesser trochanter, and proximal and distal femoral axis were positioned directly over their corresponding radio-opaque markers’ center. In addition, the brim line, obturator foramen's major and minor axes, and femoral head center were positioned. **c** Landmarks for the calibration, navigation and verification images: ellipse to match the opening of the cup (acquired first), then ipsilateral teardrop using the marker, brim line as well as obturator foramen’s major and minor axes. **d** Landmarks for trial and final images: ellipse to match the opening of the cup (acquired first), then same landmarks as the hip reference image (**b**) but with the cup center instead of the femoral head center. Markers were used to validate the accuracy of the RA-THA platform, and are not part of the clinical use of the system

Once the preparation steps were performed (Fig. 2), a reference image of the leveled pelvis (i.e., symmetrical obturator foramina) was acquired. A photo of this image on the C-arm monitor was captured using the ROSA^®^ Tablet, and the landmarks were positioned (Fig. 3a). A hip reference image was then acquired (C-arm translation) and the landmarks were positioned (Fig. 3b). The femoral head resection and reaming was performed using manual instrumentation. The cup inserter was positioned within the joint space, and the instrument was connected to the robotic arm. Two additional fluoroscopic images were acquired, and the automatically detected landmarks were reviewed and confirmed to perform a robotic registration. The robotic arm was then moved to targeted inclination and version angles using the displayed values on the screen, and the cup was impacted until it was fully seated (the robotic arm maintains target orientation during impaction). A fluoroscopic image of the hip was then acquired (verification image), and automatically detected landmarks were reviewed and confirmed (Fig. 3c).

The femoral canal was then prepared using manual instrumentation, and a trial construct was inserted and reduced. A fluoroscopic image of the hip was then acquired (trial image), and automatically detected landmarks were reviewed and confirmed (Fig. 3d). In the trial and validation panel, the LLD measurement is displayed for the selected implant components, as well as projected values for all compatible component combinations. Once the final femoral components were implanted, a fluoroscopic image was acquired (final image), and automatically detected landmarks were reviewed and confirmed on the tablet (Fig. 3d). Surgeons were able to repeat trial and final images with different components to reach the goal of 0 mm of LLD.

### Processing of CMM acquisitions

**Fig. 4:**
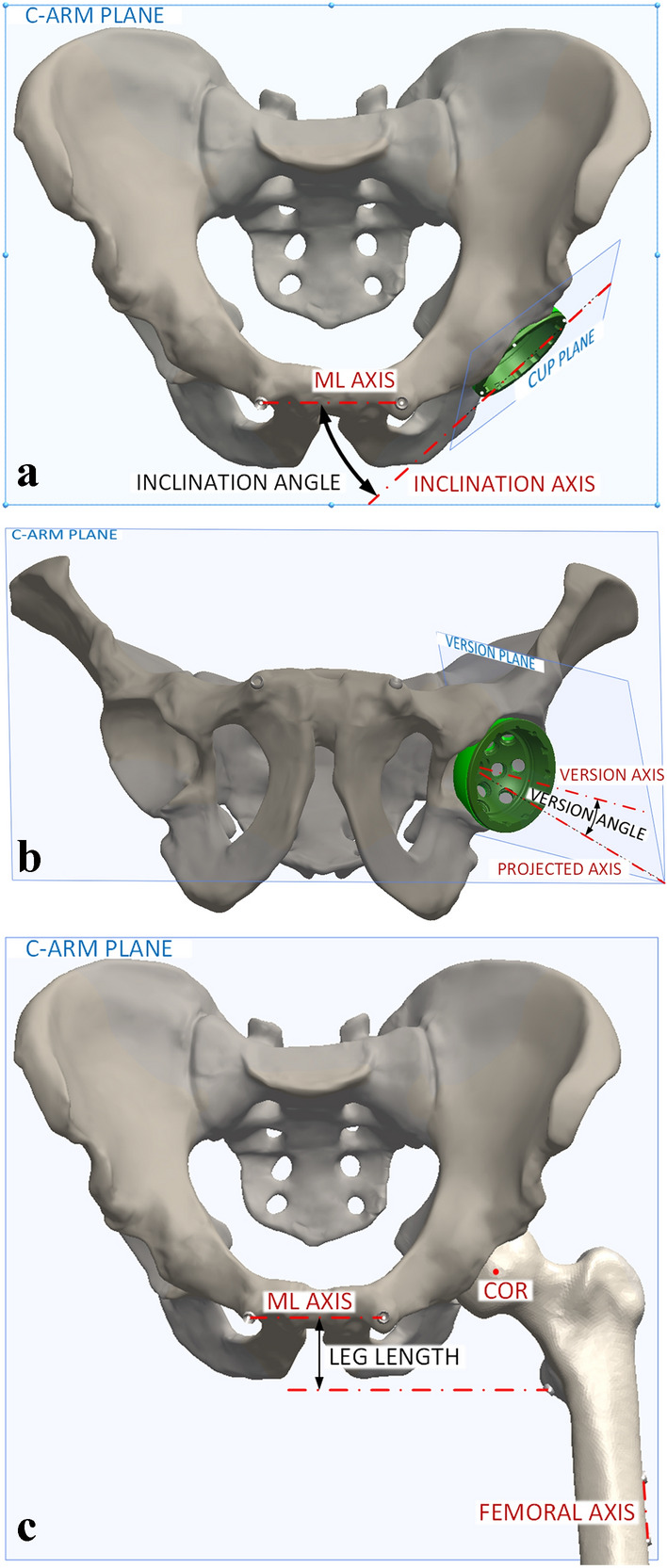
Measurement of acetabular component orientation (inclination/version) and LLD. **a** The inclination angle was determined in the C-arm detector plane, as the acute angle between the mediolateral (ML) axis (created with the ipsilateral and contralateral teardrop markers) and the inclination axis (intersection of the C-arm and cup planes). **b** The version angle was determined in the version plane (plane normal to the C-arm plane, passing through the version axis, which is normal to the cup plane), as the acute angle between the version axis and the projected axis (intersection of the C-arm and version planes). **c** The leg length was determined in the C-arm plane, as the perpendicular distance between the ML axis and the lesser trochanter marker. To determine the LLD, the post-operative and pre-operative hips were coregistered using the femoral center of rotation, and the angle in the C-arm plane between the ML axis and femoral axis (created with the proximal and distal femoral axis markers). The LLD was the difference between the post-operative and pre-operative measurements

The processing of CMM acquisitions was performed using a computer-aided design (CAD) software (SolidWorks 2018 SP4.0, Dassault Systèmes, Waltham MA, USA). The method was validated using a precise 3D-printed jig and the accuracy was determined as 0.11° ± 0.08°, 0.12° ± 0.08° and 0.22 ± 0.24 mm [mean absolute error (MAE) ± standard deviation (SD)], for the inclination, version, and LLD, respectively. Triplicates of each CMM acquisition were verified for outliers, and non-outlier points were averaged and imported into the CAD software to perform accuracy measurements (acetabular component orientation (inclination, version) and LLD; Fig. 4).

### Statistical analysis

The sample size was determined a priori using the percentage of cases within the Lewinnek safe zone. Without data on the current system, the percentage of cases within the safe zone was projected at 95%. As for the manual group, it was calculated at 73.6% based on the literature data [8, 13, 17–19]. Using the one proportion sample size calculation method (*α* = 0.05; power 80%), a minimum sample size of 24 hips per group was determined.

After testing for data normality, descriptive statistics were calculated: MAE, SD, 95% confidence interval (CI), absolute min and max, percentage of cases within a safe zone. Group comparison of the MAE, variance and percentage of cases within a safe zone was performed using paired Student *t* test, *F* test and Fisher’s exact test, respectively, with significance determined at *p* < 0.05 (SAS version 9.4).

## Results

### Acetabular component orientation

**Fig. 5:**
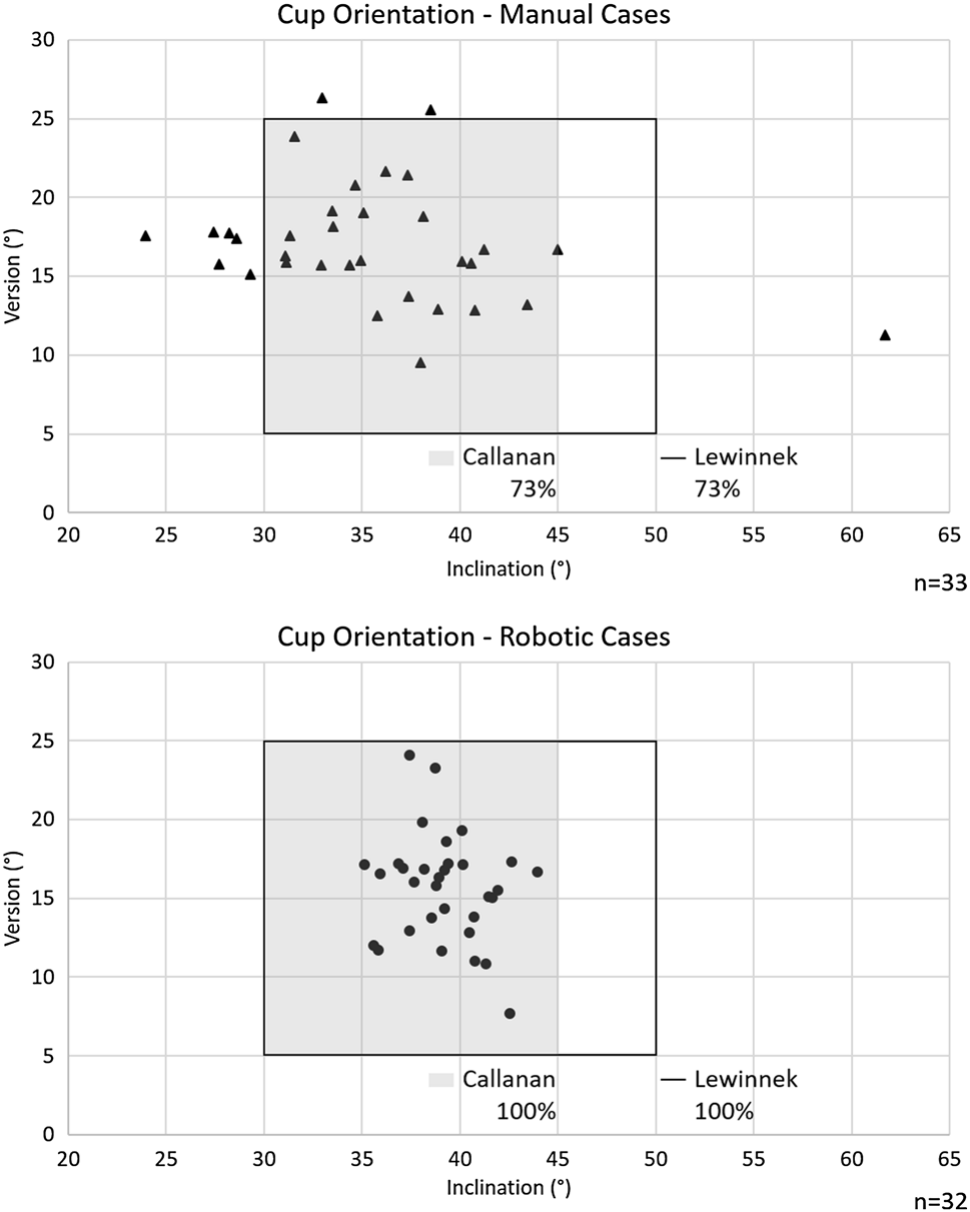
Scatterplots of manual (top) and robotic (bottom) cases within the Lewinnek and Callanan safe zones. The acetabular component orientation is significantly more reproducible (fewer outliers) in the robotic group compared to the manual group (*p* = 0.002)

The accuracy of cup inclination was significantly improved in the robotic group compared to the manual group (*p* < 0.001; Table 2). Moreover, the variance was significantly lower (i.e., fewer chances of an outlier) in the robotic group (*p* < 0.001; Table 2). The accuracy and variance of cup version did not differ significantly between the groups (*p* > 0.05; Table 2), even though the robotic group had a better accuracy (MAE of 2.6° vs 3.3°) and a lower variance (2.3° vs 2.8°) compared to the manual group. The percentage of cases within the Lewinnek and Callanan safe zones was significantly higher (fewer outliers) for the robotic group compared to the manual group (100% vs 73% *p* = 0.002; Fig. 5).

**Table 2:** Accuracy of reproducing the intraoperative plan

Parameter	Mean |Δ| ± SD [CI 95%]		Paired *t* test*	F test**	|Min|, |Max|	
	Manual	Robotic	*p* value	*p* value	Manual	Robotic
Sample size	33	32^a^			33	32^a^
Inclination (°)	6.4 ± 4.9 [4.6–8.1]	1.8 ± 1.3 [1.4–2.3]	** < 0.001**	** < 0.001**	0.1, 21.7	0.3, 4.6
Version (°)	3.3 ± 2.8 [2.3–4.3]	2.6 ± 2.3 [1.8–3.4]	0.198	0.206	0.1, 11.3	0.0, 9.0
LLD (mm)	3.5 ± 4.1 [2.1–5.0]	2.3 ± 2.4 [1.4–3.1]	0.105	**0.003**	0.0, 18.7	0.0, 9.4

The accuracy of inclination, version and leg length discrepancy (LLD) was determined as the mean absolute error (Mean |Δ|) between the values obtained from the processing of CMM acquisitions and the target values

*SD* standard deviation, *CI* confidence interval, |*Min*| absolute minimum value, |*Max*| absolute maximum value

^a^Incorrect CMM acquisition of the cup rim plane for one case (*n* = 32 for inclination and version), and lesser trochanter marker not acquired for one case (*n* = 32 for LLD)

*Group comparison of the mean absolute error (Mean |Δ|) using Student *t* test

**Group comparison of the variance using *F* test; significant *p* values (*p* < 0.05) in bold

### Leg length discrepancy (LLD)

The ability to equalize LLD did not differ significantly between the groups (*p* > 0.05; Table 2), even though the robotic group had a better accuracy (2.3 mm vs 3.5 mm) compared to the manual group. The variance of LLD was significantly lower in the robotic group (*p* = 0.003; Table 2), indicating fewer outliers, compared to the manual group.

## Discussion

This study presents the results of a novel, CT-free and pin-less robotic-assisted platform for primary THA. When compared to a control group of fluoroscopic-guided manual THA performed by high-volume arthroplasty surgeons, the RA-THA system demonstrated accurate and reproducible component positioning and restoration of key biomechanical parameters. Clinical advantages of this novel RA-THA system include: no need for special imaging (lower cost), no bone trackers, and no change to the surgeon’s individual workflow or surgical approach.

It is important to highlight the user group in this matched-pair study: a group of high-volume hip arthroplasty surgeons (range 175–810 procedures/year) with a majority (12/14; 85%) experienced with fluoroscopy use during mTHA. Prior studies examining RA-THA applications in general have not included a large control group of highly experienced THA surgeons, and instead relied largely on single-surgeon study designs for comparisons between RA-THA and mTHA [4, 8, 9, 11, 12, 14, 20–22].

The CT-free robotic-assisted surgery platform, using fluoroscopic images alone, demonstrated more accurate acetabular cup positioning, when compared to the mTHA procedures, with respect to cup inclination and placement within the Lewinnek [23] and Callanan [24] safe zones. Precise acetabular component placement mitigates the risk of hip instability, while avoiding impingement and restriction of the range of motion [23, 24], but achieving accurate placement can be technically challenging. Surgeons with variable experience must account for potentially distorted or obscured bony landmarks, variation of intraoperative pelvic tilt and positioning, the limited accuracy of conventional alignment guides [25, 26], and body habitus factors like obesity [27]. The RA-THA system in this report offers the ability to reduce outliers in acetabular component orientation.

In a meta-analysis of studies that reported rates of placement within the safe zones of Lewinnek and Callanan for inclination and version (semi-active robots) [28], RA-THA was associated with more accurate cup positioning than mTHA (Lewinnek: OR 9.24, 95% CI [6.15, 13.89], *p* < 0.00001; Callanan: OR 7.03, 95% CI [5.12, 9.65], *p* < 0.00001). For the cup positioning, our study demonstrated that RA-THA was more accurate than mTHA for cup inclination (no difference for version), while both parameters did not show a significant difference in that same meta-analysis [28]. Only one matched-pair cadaveric study, using a semi-active RA-THA system versus mTHA in only six specimens, found increased accuracy with respect to cup version [21].

Radiographic LLD remains a significant source of potential litigation where “operator error” accounted for the second most common cause of malpractice claims within the British National Health Service between 2002 and 2007 [29]. In a meta-analysis of studies that compared the LLD between RA-THA and mTHA [28], RA-THA was found to result in significantly lower LLD compared to mTHA (MWD: − 1.24 mm, 95% CI [− 2.15 mm, − 0.33 mm], *p* = 0.008)*.* While our study did not find a significant difference between groups, the mean absolute error of LLD for RA-THA was lower than mTHA. It is also well under the threshold most patients can tolerate (< 10 mm; [30]), and consistent with values reported in the robotic literature. However, the RA-THA group in this study demonstrated significantly more reproducible LLD (fewer outliers) compared to mTHA (*p* = 0.003).

In studying this RA-THA system that requires only intraoperative fluoroscopic 2-D images, we sought to compare the THA systems that utilize fluoroscopic images for data acquisition and intraoperative guidance. Unfortunately, these commercially available systems have limited published studies to date. One study using the Velys™ Hip Navigation system (Depuy Synthes, Warsaw IN, USA) demonstrated intraoperative efficiencies but no difference in LLD in a retrospective case review [31]. No information was provided on other radiographic measurements like the accuracy of cup implantation. In a study of a single-plane intraoperative fluoroscopic measurement system (RadLink; El Segundo, CA, USA) versus post-operative biplane radiographic system (3D SterEOS software, EOS Imaging, SA, Paris, France) in 48 consecutive patients in the direct anterior THA approach, the single-plane software identified two acetabular cups outside of the safe zone [32]. However, the SterEOS identified 12 (anatomic plane) and 10 (functional plane) cups outside of the safe zone. A prospective clinical study of the RadLink software with the anterior approach to THA demonstrated significant improvement in inclination but no difference in version [33]. Eighty-seven percent of the software-guided cases were within 5° of the target inclination goal of 40°, compared to 100% in this study. These software-only systems provide only descriptive analysis of fluoroscopic images, and do not allow for robotic-assistance of intraoperative component placement.

This study has several limitations. It consisted of high-volume surgeons, which may limit the generalizability of the findings: the mean number of THA procedures per surgeon in the United States, as reported in the American Joint Replacement Registry, is ≈30 THA/year [34]. The mean THA annual procedural volume for surgeons in our study was 323 cases. However, adding lower volume surgeons would have likely increased the significance of certain measured parameters, as the use of robotic-assistance reduced outliers even in our group of highly experienced hip surgeons, for example, with respect to acetabular safe zones. We did not report on offset as one of our primary study measures, and this parameter should be included in future study of this technology. Finally, we chose to utilize the safe zones described by Lewinnek and Callanan as measures of the ideal acetabular implant orientation. More recently, the value of these zones in preventing hip instability has been questioned [35]. Nevertheless, these safe zones remain a measure of the preoperatively planned target orientation in most of the included literature and can, therefore, be used as a benchmark for the accuracy of implant positioning compared to a pre-operative plan.

Future directions for study include understanding this novel RA-THA system with respect to varying component placement from a standardized target (e.g., 40°/15° for inclination/version). This may inform the benefits of RA-THA technology to match target values with certain clinical relevance, such as targets that may be chosen to match pre-operative analysis of spino-pelvic motion. The learning curve surrounding this fluoroscopic-based robotic system should be studied, as there is promise for accelerating the interpretation and analysis of fluoroscopic images utilizing this current technology [36]. Future study should also include comparator surgeons with little fluoroscopic experience, as well as a direct comparison to mTHA with no fluoroscopic confirmation of component position intraoperatively. For both novice and experienced surgeons, the use of fluoroscopy may further increase the accuracy of mTHA procedures [37]. Overall operating time and efficiency of surgical workflow should be another target of future study, along with associated costs, intraoperative work-times, and the clinical outcomes [38] between commercially available robotic systems that require a pre-operative CT scan and this system that requires only 2-D intraoperative fluoroscopic imaging.

## Conclusion

The novel CT-free and pin-less robotic-assisted platform, using fluoroscopic images alone, demonstrated more accurate acetabular cup positioning, when compared to the mTHA procedures performed by high-volume hip surgeons (naive to this RA-THA system), with respect to cup inclination and placement within the Lewinnek and Callanan safe zones. This study supports the clinical use of this system for primary THA. Future study must incorporate economic factors, clinical and patient-centric outcomes, and other radiographic parameters in controlled studies in large sample sizes.

## Data Availability

All the data generated during this study are included in this published article. Additional information can be requested from the corresponding author.
