# Correction to: Improved accuracy and fewer outliers with a novel CT-free robotic THA system in matched-pair analysis with manual THA

**DOI:** 10.1007/s11701-021-01347-9

**Published:** 2021-12-06

**Authors:** Atul F. Kamath, Sridhar M. Durbhakula, Trevor Pickering, Nathan L. Cafferky, Trevor G. Murray, Michael A. Wind, Stéphane Méthot

**Affiliations:** 1 Orthopaedic and Rheumatologic Institute, Cleveland Clinic, 9500 Euclid Avenue, A40, Cleveland, OH 44195 USA; 2 Washington Joint Institute, Bethesda, MD 20817 USA; 3 Mississippi Sports Medicine and Orthopaedic Center, Jackson, MS 39202 USA; 4 Vail Summit Orthopaedic Foundation, Vail, CO 81657 USA; 5 OrthoVirginia, Richmond, VA 23235 USA; 6 Zimmer CAS, Montréal, QC H3C-2N6 Canada

## Correction to: Journal of Robotic Surgery 10.1007/s11701-021-01315-3

The article Improved accuracy and fewer outliers with a novel CT-free robotic THA system in matched-pair analysis with manual THA, written by Atul F. Kamath, Sridhar M. Durbhakula, Trevor Pickering, Nathan L. Cafferky, Trevor G. Murray, Michael A. Wind Jr. and Stéphane Méthot, was originally published electronically on the publisher’s internet portal on 28 October 2021 without open access. With the author(s)’ decision to opt for Open Choice the copyright of the article changed on 22 November 2021 to © The Author(s) 2021 and this article is licensed under a Creative Commons Attribution 4.0 International License, which permits use, sharing, adaptation, distribution and reproduction in any medium or format, as long as you give appropriate credit to the original author(s) and the source, provide a link to the Creative Commons licence, and indicate if changes were made. The images or other third party material in this article are included in the article’s Creative Commons licence, unless indicated otherwise in a credit line to the material. If material is not included in the article's Creative Commons licence and your intended use is not permitted by statutory regulation or exceeds the permitted use, you will need to obtain permission directly from the copyright holder. To view a copy of this licence, visit http://creativecommons.org/licenses/by/4.0/.

The original article has been corrected.

